# Supplemental Selenium and Boron Mitigate Salt-Induced Oxidative Damages in *Glycine max* L.

**DOI:** 10.3390/plants10102224

**Published:** 2021-10-19

**Authors:** Mira Rahman, Khussboo Rahman, Khadeja Sultana Sathi, Md. Mahabub Alam, Kamrun Nahar, Masayuki Fujita, Mirza Hasanuzzaman

**Affiliations:** 1 Department of Agronomy, Faculty of Agriculture, Sher-e-Bangla Agricultural University, Dhaka 1207, Bangladesh; mirarahman73@gmail.com (M.R.); khussboorahman1305594@sau.edu.bd (K.R.); sathikhadeja1405945@sau.edu.bd (K.S.S.); shamim1983@sau.edu.bd (M.M.A.); 2 Department of Agricultural Botany, Faculty of Agriculture, Sher-e-Bangla Agricultural University, Dhaka 1207, Bangladesh; knahar84@yahoo.com; 3 Laboratory of Plant Stress Responses, Faculty of Agriculture, Kagawa University, Miki-cho, Kita-gun, Kagawa 761-0795, Japan

**Keywords:** abiotic stress, AsA-GSH pathway, methylglyoxal, micronutrient, osmoregulation, reactive oxygen species, trace elements

## Abstract

The present investigation was executed with an aim to evaluate the role of exogenous selenium (Se) and boron (B) in mitigating different levels of salt stress by enhancing the reactive oxygen species (ROS) scavenging, antioxidant defense and glyoxalase systems in soybean. Plants were treated with 0, 150, 300 and 450 mM NaCl at 20 days after sowing (DAS). Foliar application of Se (50 µM Na_2_SeO_4_) and B (1 mM H_3_BO_3_) was accomplished individually and in combined (Se+B) at three-day intervals, at 16, 20, 24 and 28 DAS under non-saline and saline conditions. Salt stress adversely affected the growth parameters. In salt-treated plants, proline content and oxidative stress indicators such as malondialdehyde (MDA) content and hydrogen peroxide (H_2_O_2_) content were increased with the increment of salt concentration but the relative water content decreased. Due to salt stress catalase (CAT), monodehydroascorbate reductase (MDHAR), dehydroascorbate reductase (DHAR), glyoxalase I (Gly I) and glyoxalase II (Gly II) activity decreased. However, the activity of ascorbate peroxidase (APX), glutathione reductase (GR), glutathione peroxidase (GPX), glutathione *S*-transferase (GST) and peroxidase (POD) increased under salt stress. On the contrary, supplementation of Se, B and Se+B enhanced the activities of APX, MDHAR, DHAR, GR, CAT, GPX, GST, POD, Gly I and Gly II which consequently diminished the H_2_O_2_ content and MDA content under salt stress, and also improved the growth parameters. The results reflected that exogenous Se, B and Se+B enhanced the enzymatic activity of the antioxidant defense system as well as the glyoxalase systems under different levels of salt stress, ultimately alleviated the salt-induced oxidative stress, among them Se+B was more effective than a single treatment.

## 1. Introduction

Abiotic stress or environmental stress is not a sole entity. Under the umbrella of abiotic stress, it comprises all types of hostile environmental conditions that a plant may face in nature [1]. Salt stress is a major abiotic stress. Salinity threatens the productivity of plants by negatively affecting the biochemical, physiological and molecular features of the plants [2]. Because of inappropriate management and climate change, the saline-affected area has been increasing more than before in arid, semi-arid and coastal areas, along with other types of land [3]. Worldwide, about 20–50% of irrigated land areas are affected by salt [3]. The alarming issue is by 2050 up to 50% of agricultural land is expected to be affected by salinity [2].

In the soil solution, sodium chloride (NaCl) and sodium sulfate (Na_2_SO_4_) are the most available soluble salts. An increase in salinity level, in most of the cases, indicates mainly an increase in Na^+^ and Cl^−^ concentration. Both Na^+^ and Cl^−^ ions produce critical conditions for plant survival, but between them, Cl^−^ is more dangerous [4]. Salinity primarily creates osmotic stress and ionic toxicity. Osmotic stress occurs due to the accumulation of a higher concentration of salt ions in the root zone. Osmotic stress hinders the uptake of water and nutrient of the plants and ultimately causes stomatal closure, reduction in cell expansion and division. In the later stage, a higher accumulation of salt inside the cells and tissues induces ionic toxicity, disruption of ion homeostasis, alteration of cellular functions, premature senescence, and in extreme condition plant death. Salinity-induced osmotic and ionic stresses are responsible for the overproduction of reactive oxygen species (ROS) [5]. Therefore, an excess concentration of ROS in plants induces deleterious oxidative stress, which causes oxidation of plant cell components (lipid, protein, nucleic acid, etc.), along with the cell organelles and membranes which also disrupts the redox homeostasis [6]. The overproduction of ROS such as superoxide radical (O_2_^•−^), hydrogen peroxide (H_2_O_2_), hydroxyl radical (^•^OH) etc. is needed to be stopped to protect the plants from oxidative damage and to regulate the proper physiological and biochemical activities. Plants maintain the balance between formation and detoxification of ROS by an antioxidant defense system [7]. Multiple enzymatic components of antioxidant defense system like catalase (CAT), ascorbate peroxidase (APX), monodehydroascorbate reductase (MDHAR), dehydroascorbate reductase (DHAR), glutathione reductase (GR), glutathione peroxidase (GPX), glutathione *S*-transferase (GST), and peroxidase (POD) coordinately act to control the ROS and ROS-induced oxidative stress, along with non-enzymatic components [8].

Many investigations have been carried out and attempts have been taken to mitigate the hazardous effect of salt stress on crops [9,10,11]. After a plethora of investigations, selenium (Se) a beneficial trace element (at lower concentrations) was found to be an effective one in improving growth, antioxidant defense and inducing tolerance mechanisms against salt stress [9,10,11]. Selenium acts as a plant growth regulator, stress modulator, antioxidant agent at lower concentrations but at higher concentrations, it is phytotoxic and may act as a pro-oxidant [12]. Supplementation of Se mitigates salt stress by reducing Na^+^ accumulation in plant parts, Na^+^ compartmentalization, upregulating Na^+^ and Cl^−^ ions transporter genes, chelation and boosting of the antioxidant defense system. Selenium protects plants from oxidative stress by triggering the detoxification of ROS, which is overgenerated due to salt stress [9,10,12]. Boron (B) is an essential micronutrient that actively participates in the crop growth and development process. It is associated with respiration, transportation of water, protein synthesis, sugar transport, RNA metabolism and plant hormones. Importantly, it maintains the structural integrity of bio-membranes [13]. Boron is involved with lignin synthesizing, strengthening the cell walls, and indirectly protecting cell membranes in plants [14]. Supplementation of B increased the pectin (19%) and hemicellulose (50%) content of the cell wall under oxidative stress [15]. Exogenous application of B decreased the Cl^−^ content in sugar beet under salt stress by upregulating the transportation of Cl^−^ ions [16]. Supplementation of B declined lipid peroxidation (indicated by malondialdehyde, MDA content) and hydrogen peroxide (H_2_O_2_) content under oxidative stress. Moreover, the application of B increased the antioxidant defense and decreased oxidative stress in different crops [15,17]. However, among the different combinations of treatments with or without Fe, Se+B treatment alone showed the maximum relative water content (RWC) and the minimum relative water loss, and also improved the growth parameters along with other treatments [18].

Soybean (*Glycine max* L.) is a widely cultivated legume around the world because of its versatile uses and economic importance. It is a prominent source of proteins and edible oil, it has valuable uses as food, feed and oilseed crop [19]. In soybean plants, salinity creates oxidative, osmotic and ionic stress [20]. Salt-induced osmotic, ionic and oxidative stress hampers the growth, biomass production and crucial physiological activities of soybean, ultimately productivity is negatively affected [20,21].

In previous investigations, the positive effect of Se and B in improving the salt tolerance of plants were studied in the laboratory and hydroponic conditions, mostly. However, the interaction role of Se and B, along with individual applications was rarely studied. Moreover, the role of B in methylglyoxal (MG) detoxification was hardly studied. The present investigation was conducted to study the injurious effect of the different levels of salt stress on soybean and also to study the roles of Se, B and Se+B in mitigating salt-induced oxidative stress, and enhancing the salt stress tolerance in soybean by upregulating the antioxidant defense system and MG detoxification system.

## 2. Results

### 2.1. Growth Parameters

**Figure 1:**
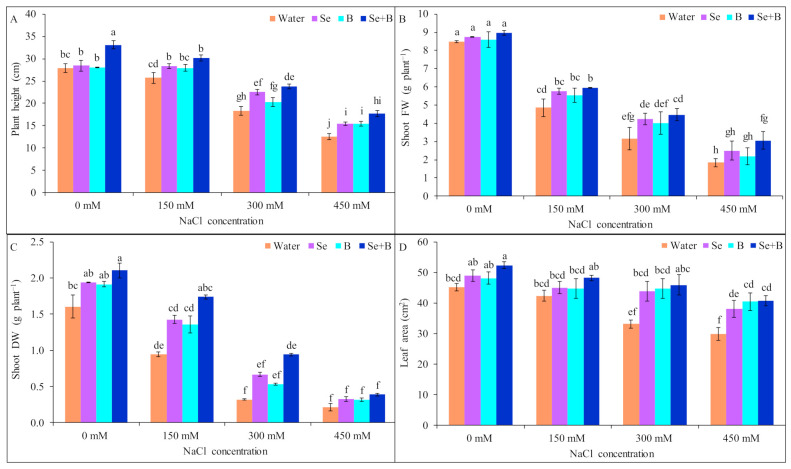
Effect of exogenous Se, B and Se+B on plant height (**A**), shoot FW (**B**), shoot DW (**C**) and leaf area (**D**) in soybean at 0, 150, 300 and 450 mM NaCl-induced salt stress. Here, Se, B and Se+B indicate 50 µM Na_2_SeO_4_, 1 mM H_3_BO_3_ and 50 µM Na_2_SeO_4_ + 1 mM H_3_BO_3_, respectively. Values in a column with different letters are significantly different at *p* ≤ 0.05 applying Fisher’s LSD test.

Upon exposure to 150 mM NaCl stress, the reduction in plant height was not significant but at 300 and 450 mM NaCl, stress plant height was reduced by 35 and 55%, respectively, in comparison to untreated control (without salt and Se, B and Se+B treatment). However, supplementation of Se and Se+B increased the plant height under all salt treatments. At 150 and 300 mM NaCl stress B alone did not increase the plant height significantly, compared to a respective only salt-treated plant without Se, B and Se+B spray. Under mild, moderate and severe salinity Se+B spray increased the plant height by 17, 30 and 39%, respectively, compared to corresponding salt-treatment alone (150, 300 and 450 mM NaCl-treated plant without Se, B and Se+B spray; Figure 1A).

Shoot fresh weight (FW) was declined by 43, 63 and 78% at 150, 300 and 450 mM NaCl stress, in comparison to untreated control. Rather than single supplementation, Se+B combinedly increased the shoot FW significantly under salt stress. Application of Se+B spray increased the shoot FW by 22, 41 and 66% at 150, 300 and 450 mM NaCl stress, respectively, in comparison to corresponding only NaCl-treated plants (Figure 1B).

Shoot dry weight (DW) was decreased by 41% at 150 mM NaCl stress, compared to untreated control. Moreover, at 300 and 450 mM NaCl stress the reduction in shoot DW plant^−1^ was statistically similar, in comparison to untreated control. However, Se and B alone did not increase the shoot DW significantly under salt stress. Combinedly Se+B increased the shoot DW plant^−1^ significantly by 85 and 93% at 150 and 300 mM NaCl stress but at 450 mM NaCl stress the increment of shoot DW was not significant, respectively, compared to respective salt treatment alone (Figure 1C).

In 150 mM NaCl-stressed plants, reduction in leaf area was not significant, compared to untreated control. Moreover, at 300 mM and 450 mM NaCl stress decrement of leaf area was statistically similar. Single supplementation of Se and B showed statistically similar results to Se+B and increased the leaf area at 300 and 450 mM NaCl stress but not at 150 mM NaCl stress. However, due to Se+B spray at 300 and 450 mM NaCl stress, leaf area was increased by 39 and 37%, respectively, compared to salt-treated plant alone (without Se, B and Se+B spray; Figure 1D).

### 2.2. Leaf Relative Water Content

**Figure 2:**
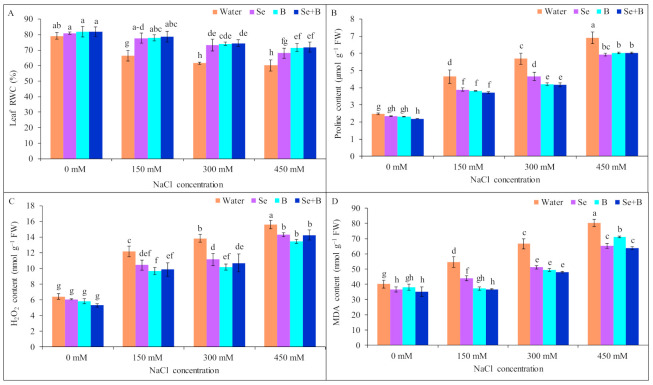
Effect of exogenous Se, B and Se+B on leaf RWC (**A**), Pro content (**B**), H_2_O_2_ content (**C**) and MDA content (**D**) in soybean at 0, 150, 300 and 450 mM NaCl-induced salt stress. Here, Se, B and Se+B indicate 50 µM Na_2_SeO_4_, 1 mM H_3_BO_3_ and 50 µM Na_2_SeO_4_ + 1 mM H_3_BO_3_, respectively. Values in a column with different letters are significantly different at *p* ≤ 0.05 applying Fisher’s LSD test.

Due to imposition of 150 mM NaCl stress leaf RWC was decreased by 19%, and at 300 and 450 mM NaCl stress the reduction in leaf RWC was statistically similar, respectively, in comparison to untreated control (without salt and Se, B and Se+B treatment). On the contrary, foliar application of Se, B and Se+B increased the leaf RWC and showed a statistically similar increment of leaf RWC under different levels of NaCl stress, compared to only salt-treated plants. Under mild, moderate and severe salinity Se+B spray increased the leaf RWC by 18, 21 and 20%, respectively, compared to corresponding salt-treatment alone (150, 300 and 450 mM NaCl-treated plant without Se, B and Se+B spray; Figure 2A).

### 2.3. Proline Content

A higher amount of proline (Pro) accumulation was observed in 150, 300 and 450 mM NaCl-stressed plants; 88, 131 and 180%, respectively, in comparison to untreated control. On the other hand, foliar-applied Se, B and Se+B diminished the Pro content in saline condition only. Moreover, the single and combined application of Se and B showed a statistically similar result in decreasing Pro content under salt stress. Application of Se+B decreased the Pro content by 20, 19 and 12% at 150, 300 and 450 mM NaCl stress, respectively, compared to corresponding only NaCl-treated plants (150, 300 and 450 mM NaCl-treated plants without Se, B and Se+B treatments; Figure 2B).

### 2.4. H_2_O_2_ Content

In salt-stressed plants, H_2_O_2_ content was increased by 40, 93 and 131% under mild, moderate and severe salinity, respectively, in comparison to untreated control plants. However, Se and B alone and combinedly showed a statistically similar result in the case of decreasing the H_2_O_2_ content under different levels of salt stress. Moreover, H_2_O_2_ content decreased due to Se+B spray by 17, 21 and 24% under mild, moderate and severe salinity, respectively, compared to corresponding only salt-treated plants (Figure 2C).

### 2.5. Lipid Peroxidation (MDA Content)

Salt-induced oxidative stress is responsible for lipid peroxidation. In order to estimate the lipid peroxidation level, MDA content is measured as a major indicator.

When subjected to salt stress MDA content was increased with the increase in the NaCl concentration, in a dose-dependent manner. Under mild, moderate and severe salinity MDA content was increased sharply by 112, 142 and 172%, respectively, compared to untreated control. On the contrary, under saline conditions, exogenous Se, B and Se+B diminished the MDA content. However, MDA content was reduced due to Se+B application by 19, 23 and 9% under mild, moderate and severe salinity, compared to respective salt treatment alone (Figure 2D).

### 2.6. Activities of Antioxidant Enzymes

**Figure 3:**
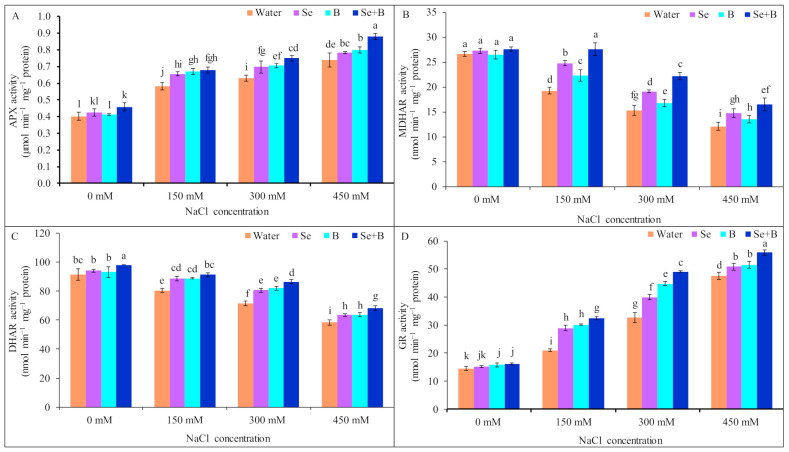
Effect of exogenous Se, B and Se+B on APX activity (**A**), MDHAR activity (**B**), DHAR activity (**C**) and GR activity (**D**) in soybean at 0, 150, 300 and 450 mM NaCl-induced salt stress. Here, Se, B and Se+B indicate 50 µM Na_2_SeO_4_, 1 mM H_3_BO_3_ and 50 µM Na_2_SeO_4_ + 1 mM H_3_BO_3_, respectively. Values in a column with different letters are significantly different at *p* ≤ 0.05 applying Fisher’s LSD test.

The activity of APX was increased by 45, 58 and 85% under mild, moderate and severe salinity, respectively, compared to untreated control (without salt and Se, B and Se+B treatment). Moreover, foliar application of Se, B and Se+B further increased the APX activity in plants under different levels of salinity, compared to only salt-treated plants. Supplementation of Se+B enhanced the activity of APX at 150 mM NaCl stress which was statistically similar to the result of Se and B alone. However, at 300 and 450 mM NaCl stress, Se+B amplified the APX activity more than the individual supplementation of Se and B, and increased the APX activity by 19 and 19%, respectively, in comparison to corresponding salt treatment alone (Figure 3A).

In response to 150, 300 and 450 mM NaCl stress, MDHAR activity was declined by 27, 42 and 54%, respectively, in comparison to untreated control. On the contrary, Se, B and Se+B treatments increased the MDHAR activity under salt stress, compared to corresponding salt treatment alone. Among them Se+B showed more increment in MDHAR activity than single supplementation. However, at 150, 300 and 450 mM NaCl stress exogenous Se+B enhanced the MDHAR activity by 43, 45 and 36%, respectively, in comparison to respective only salt-treated plants (Figure 3B).

The DHAR activity was decreased by 12, 22 and 36% at 150, 300 and 450 mM NaCl, respectively, in comparison to untreated control. However, DHAR activity was enhanced under salt stress because of Se, B and Se+B spray, compared to the respective only salt treatment (without Se, B and Se+B spray). A statistically similar enhancement was observed in the DHAR activity at 150 mM NaCl stress due to the application of Se, B and Se+B spray, compared to 150 mM NaCl stress (without Se, B and Se+B spray). The combined application of Se+B increased the DHAR activity by 21 and 16%, respectively, at 300 and 450 mM NaCl, in comparison to the corresponding salt treatment alone which was followed by individual application of Se and B (Figure 3C).

When exposed to 150, 300 and 450 mM NaCl, GR activity was increased by 45, 126 and 228%, respectively, compared to untreated control. However, exogenous supplementation of Se, B and Se+B further increased the GR activity at 150, 300 and 450 mM NaCl, compared to salt treatment alone. In combined Se+B increased the GR activity more than spraying with of Se or B alone, and due to exogenous Se+B the GR activity was accelerated by 54, 50 and 18% at 150, 300 and 450 mM NaCl, respectively, in comparison to respective only salt-treated plants (Figure 3D).

**Figure 4:**
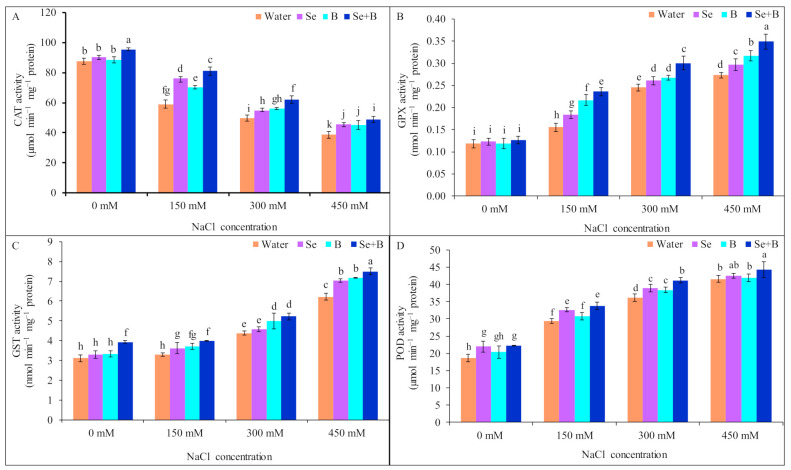
Effect of exogenous Se, B and Se+B on CAT activity (**A**), GPX activity (**B**), GST activity (**C**) and POD activity (**D**) in soybean at 0, 150, 300 and 450 mM NaCl-induced salt stress. Here, Se, B and Se+B indicate 50 µM Na_2_SeO_4_, 1 mM H_3_BO_3_ and 50 µM Na_2_SeO_4_ + 1 mM H_3_BO_3_, respectively. Values in a column with different letters are significantly different at *p* ≤ 0.05 applying Fisher’s LSD test.

All salt treatments caused a substantial reduction in the CAT activity, in comparison to untreated control. Under mild, moderate and severe salinity CAT activity was decreased by 33, 43 and 56%, respectively, compared to untreated control. On the contrary, foliar application of Se, B and Se+B enhanced the CAT activity under salt stress. Among the foliar supplementations, Se+B showed a higher increment in CAT activity than Se or B alone. Moreover, application of Se+B increased the CAT activity by 38, 25 and 27% under mild, moderate and severe salinity, respectively, in comparison to respective salt treatment alone (Figure 4A).

In NaCl-treated plants, GPX activity was enhanced by 33, 108 and 125%, respectively, in comparison to untreated control. Moreover, foliar supplementation of Se, B and Se+B further stimulated the GPX activity under different levels of salinity. Among the treatments, under salt stress Se+B increased the GPX activity more than Se or B alone. Comparing with corresponding salt-treated plants (without Se, B and Se+B spray), GPX activity was increased by 66, 20 and 29%, respectively, in Se+B-treated plants under mild, moderate and severe salinity (Figure 4B).

Between untreated control plants and 150 mM salt-treated plants, no significant difference in GST activity was recorded. Moreover, at 300 and 450 mM salt stress GST activity was increased by 41 and 111%, in comparison to untreated control. However, at 150, 300 and 450 mM salt stress, application of Se+B further accelerated the GST activity by 21, 19 and 21%, respectively, compared to the respective only salt treatment. Although Se+B showed higher GST activity than an individual spray, but at 150 and 300 mM NaCl stress, B spray showed a similar increase in GST activity as Se+B spray, compared to only salt treatment (Figure 4C).

When plants were exposed to 150, 300 and 450 mM salt stress, POD activity was increased by 58, 95 and 124%, respectively, in comparison to untreated control. Application of Se+B increased the POD activity by 15, 14 and 7% at 150, 300 and 450 mM salt stress, respectively, compared to respective salt treatment alone. Moreover, no significant increment in POD activity was observed at 150 and 450 mM NaCl stress because of single supplementation of B. Single supplementation of Se caused a statistically similar increment of POD activity as Se+B at 150 and 450 mM NaCl stress, compared to corresponding salt treatment alone (Figure 4D).

### 2.7. Activities of Glyoxalase Enzymes

The activity of Gly I and Gly II showed reverse relation with the salt concentration, Gly I and Gly II activity decreased with the gradual increase in the salt concentration.

**Figure 5:**
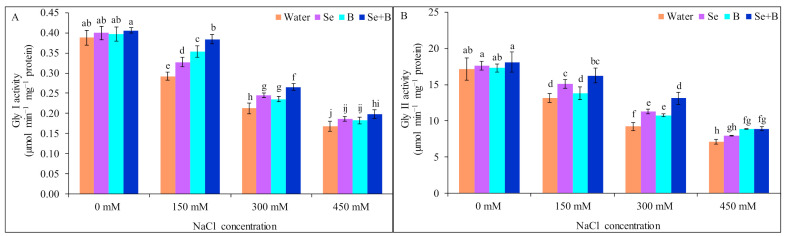
Effect of exogenous Se, B and Se+B on glyoxalase system: Gly I activity (**A**) and Gly II activity (**B**) in soybean at 0, 150, 300 and 450 mM NaCl-induced salt stress. Here, Se, B and Se+B indicate 50 µM Na_2_SeO_4_, 1 mM H_3_BO_3_ and 50 µM Na_2_SeO_4_ + 1 mM H_3_BO_3_, respectively. Values in a column with different letters are significantly different at *p* ≤ 0.05 applying Fisher’s LSD test.

Under mild, moderate and severe salinity Gly I activity was declined by 26, 46 and 56%, respectively, in comparison to untreated control. On the contrary, foliar application of Se and B alone significantly increased the Gly I activity under mild and moderate salinity but not at severe salinity. Moreover, combined application of Se+B enhanced the Gly I activity by 31, 29 and 18% under mild, moderate and severe salinity, respectively, in comparison to corresponding salt treatment alone, followed by an individual spray of Se and B (Figure 5A).

In response to mild, moderate and severe salinity the activity of Gly II was declined by 24, 46 and 59% in 150, 300 and 450 mM NaCl-treated plants, respectively, in comparison to untreated control (without salt and Se, B and Se+B treatment). At 150 and 300 mM NaCl stress foliar application of Se+B increased the Gly II activity by 24 and 42%, respectively, compared to respective salt-treatment alone. Moreover, at 450 mM salt stress B alone and combined supplementation of Se+B showed a similar result, in the case of increasing the Gly II activity, respectively, in comparison to corresponding only salt-treated plant (Figure 5B).

## 3. Discussion

Salt stress has a detrimental effect on the growth, development and physiological activities of soybean [20,21]. In combating salt stress, exogenous protectants (micronutrients, trace elements, osmoprotectants, phytohormones, polyamines and antioxidants) showed promising results [4]. Supplementation of beneficial trace elements like Se improves antioxidant defense, and stress tolerance in plants [12,22]. The application of micronutrients increases plant stress tolerance by upregulating physiological activities and by improving the antioxidant defense system [23].

In response to salt stress shoot growth stunts [5]. Akram et al. [24] reported salt-induced plant height reduction in different genotypes of soybean. Salinity decreased the plant height, shoot FW and shoot DW in soybean along with other growth parameters [25]. Wu et al. [26] investigated that salt stress reduced the leaf area. Due to NaCl stress, plant height, DW and leaf area were reduced in two soybean cultivars [27]. Plant height, plant FW, plant DW and leaf area were decreased in NaCl-stressed soybean plants [28]. As salt stress imposes both ionic and osmotic stress in the plant, it negatively affects the cell division and cell elongation process as well as normal cell functioning, ultimately hinders the plant growth [4]. Foliar application of Se, at low concentration, enhanced the plant height of wheat under salt stress [29]. Application of Se increased the growth and shoot DW under salt stress [9]. Foliar applied Se enhanced the leaf area of cowpea under salt stress [30]. Boron is a crucial factor for the processes like respiration, synthesis of proteins, transportation of sugars and carbohydrate metabolism. Importantly, growth hormone-like IAA is also associated with B [13]. Ullah et al. [31] found that B aided in cell division of the actively growing region, especially the region near the shoot and root tips. However, Se+B spray reverted the negative effect of salt on growth parameters, in the present study.

Higher NaCl concentration in the soil solution of the root zone decreases the water potential and also hinders the water uptake through the root [5]. Moreover, a higher accumulation of Na^+^ and Cl^−^ inside the cell also restricts the water uptake. Therefore, the plant faces physiological drought (osmotic stress) under salt stress [5]. Many researchers investigated that salt stress reduced the RWC in different crops [10,29,30]. Due to NaCl-induced osmotic stress, the RWC of the soybean plants decreased, in the current study. However, supplementation of Se improved the RWC of plants, in previous studies [10,29,30]. Upon exposure to 300 mM NaCl stress foliar-applied B reduced the concentration of Cl^−^ ion in xylem sap, thus improved the water uptake [16].

Higher accumulation of osmolytes occurs to protect the plant cells from NaCl-induced dehydration [32]. Salt stress-induced higher accumulation of Pro was observed in soybean and other crops [20,33,34]. With the increment of the NaCl concentration, Pro accumulation increased in NaCl-stressed soybean plants of the present study to protect the cell from osmotic stress by maintaining the osmotic pressure of the cell. Several previously published research articles denoted that Se and B have the potential to regulate the accumulation of osmoprotectants in plants. Selenium is involved in regulating osmoprotectants and secondary metabolites [12]. Selenium supplementation regulated the Pro content and osmotic pressure which helped in enhancing the water translocation to the shoot and ultimately increased RWC under salt stress [10]. Supplementation of B reduced the leaf Pro content under salt stress and enhanced the glycine betaine content [35]. In the present study, B improved the water uptake (RWC increased) but decreased Pro content. Selenium and B may have a role in the enhancement of the other osmolytes rather than Pro like glycine betaine, glutathione, soluble sugars [35,36,37] which acted to confer osmoprotection and improved the water content. Similarly, to our findings, supplementation of Se+B decreased the Pro content and increased the RWC under NaCl stress in stevia [18]. Boron-induced regulation of pyrroline-5-carboxylate synthetase (*P5CS*) and proline dehydrogenase (*PDH*) genes and the activities of P5CS and PDH were involved in modulating Pro accumulation in *Brassica napus* [38]. Selenium applications regulated transcript levels of genes *P5CS2* and *PDH* in fragrant rice [39]. This evidence strongly supports the osmoprotective roles of B and Se.

Salt-induced osmotic stress, ionic stress, nutrient imbalance, disrupted ion homeostasis and excessive generation of ROS, altogether threaten the antioxidant defense system of plants. However, salt-induced oxidative stress deteriorates the cell membrane, cellular organelles, cellular components (proteins, lipids, carbohydrates, etc.) and nucleic acids (DNA and RNA), and may also cause programmed cell death [2]. As H_2_O_2_ is a ROS, overproduction of H_2_O_2_ results in higher lipid peroxidation which is indicated by higher MDA content [22]. In previous investigations, salt stress was responsible for the overgeneration of H_2_O_2_ and MDA content in different soybean genotypes [20,24,40]. In the present study, H_2_O_2_ content, as well as MDA content, increased with the increase in the NaCl concentrations. Supplementation of Se protected plants from salt-induced oxidative stress by triggering the detoxification of ROS, which were generated due to salt stress [11]. Moreover, by upregulating the antioxidant defense system, Se diminished the MDA content and membrane damage under salt stress [9]. Boron plays a protective role against excessive ROS by maintaining membrane integrity, metabolic activity and enzymatic activity [13]. Supplementation of B decreased the H_2_O_2_ content and as well as MDA content under salt stress [41]. Foliar applied B upregulated the Cl^−^ transportation and protected plants from salt-induced oxidative stress [16]. However, Se and B alone and in combined alleviated the salt-induced oxidative stress, in the present investigation which was indicated by the reduced oxidative stress indicators (H_2_O_2_ and MDA content) under salt stress due to Se, B and Se+B spray.

In the antioxidant defense system four key enzymes viz. APX, MDHAR, DHAR and GR of the ascorbate-glutathione (AsA-GSH) pathway are crucial for enhancing stress tolerance as well as in minimizing the stress-induced oxidative damages, by detoxification of the ROS [8]. Moreover, APX is involved in direct ROS scavenging. In chloroplast, APX is the only ROS scavenger enzyme, as CAT is absent in chloroplast. Moreover, APX and GR are the major ROS detoxifier and also maintain redox homeostasis [7]. In soybean plants, the APX and GR activity was further increased with the increase in the salt concentration but MDHAR and DHAR activity were decreased under salt stress, in the present study. Application of Se increased the APX, MDHAR, DHAR and GR activity under salt stress [22]. Supplementation of B under salt stress increased the enzymatic activity [41]. The combined application of Se+B was more effective than individual Se and B spray in enhancing the activity of MDHAR and GR under salt stress.

Catalase produces H_2_O and O_2_ by direct dismutation of H_2_O_2_. Thus, CAT plays a major role in ROS detoxification under abiotic stress [7]. In previous studies, several scientists observed lower CAT activity in response to different levels of salt stress [33,42]. Similarly, in the present study, lower CAT activity was observed with the increment of the salt concentration, compared to untreated control. Previously, in various crops supplementation of Se at lower concentration increased the CAT activity under salt stress [10,29,43,44]. Application of B also increased the CAT activity under salt stress [41]. However, exogenous application of Se alone and with B, boosted the CAT activity in the salt-treated plant. Higher CAT activity along with lower H_2_O_2_ content under salt stress due to Se, B and Se+B spray indicated that Se and B alone and combinedly enhanced the antioxidant defense against salt-induced oxidative stress by increasing the ROS scavenging. The GPX enzymes are pivotal for cell protection and detoxification under oxidative stress. Selenium enhances the activity of GPX enzymes as it is a cofactor of GPX enzymes [45,46]. Along with peroxide breakdown, hormone biosynthesis and stress signaling GST is involved in amplifying the activity of GPX. The GPX enzymes scavenge H_2_O_2_ by utilizing GSH [7]. Upon exposure to salt stress, increased activity of GPX and GST was reported by several researchers [22,34,47]. However, GST and GPX activity increased in response to salt stress, in the present study. Foliar applied Se increased the GST and GPX activity in rapeseed under salt stress [22]. Application of B at high concentration increased the GPX activity [48]. In the current study, POD activity was increased under different levels of salinity, compared to untreated control. Likewise, in oat seedlings, the activity of POD was increased at 100 mM salt stress, in comparison to control [49]. The activity of POD was increased under salt stress in common beans [43]. Similarly, the POD activity was enhanced in maize under salinity [44]. Moreover, the application of Se at low concentration further increased the POD activity under salt-induced oxidative stress [44]. Under oxidative stress application of B also increased the POD activity [17].

Methylglyoxal is a by-product of the glyoxalase system which is a highly reactive and cytotoxic compound. The enzymes of the glyoxalase system, viz. Gly I and Gly II transform MG into nontoxic compounds. In the glyoxalase system, MG detoxification occurs in two steps. First, Gly I converts MG into *S*-D-lactoyl-glutathione by utilizing GSH. In last step, *S*-D-lactoyl-glutathione is converted into D-lactate by Gly II [8]. The activity of Gly I and Gly II decreased upon exposure to salt stress, in previous investigations [22,42]. Lower Gly I and Gly II activity under salt stress was observed, in our study which indicated that MG detoxification was not sufficient enough under salt stress. However, Se supplementation enhanced MG detoxification by amplifying the Gly I and Gly II activity under NaCl stress [22]. In the present study, in comparison to only salt-treated plants at 150 and 300 mM NaCl stress Se+B resulted in higher Gly I and Gly II activity than Se or B alone which means the higher MG detoxification under salt stress.

**Figure 6:**
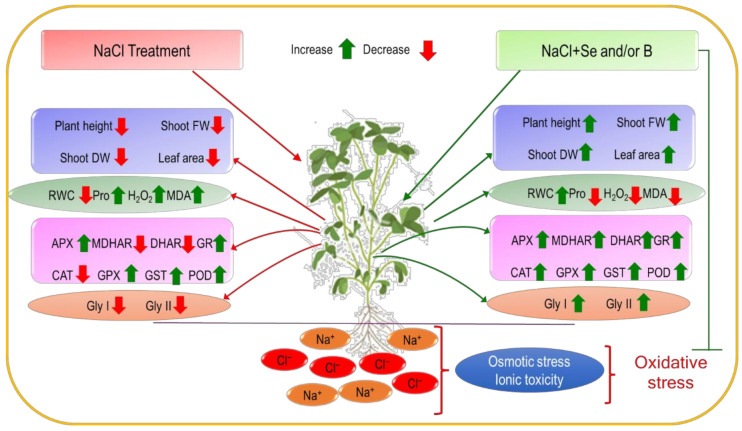
Schematic presentation of the protective roles of exogenous Se and B under salt stress.

In summary, upon exposure to different levels of NaCl-induced salt stress leaf RWC decreased along with growth parameters, but Pro content and oxidative stress indicators (H_2_O_2_ and MDA content) increased. On the contrary, Se and B alone and combined improved the growth parameters, leaf RWC and decreased the ROS accumulation which was clear by the reduced H_2_O_2_ and MDA content (lipid peroxidation), and also decreased the Pro content when subjected to salt stress. All NaCl treatments decreased the MDHAR, DHAR and CAT activity and enhanced the APX, GR, GPX, GST and POD activity. However, exogenous Se, B and Se+B spray enhanced the enzymatic activities (APX, MDHAR, DHAR, GR, CAT, GPX, GST, POD) as a part of the antioxidant defense system under salt stress. Along with other enzymatic activities, the activity of Gly I and Gly II were also increased due to single and combined application of Se and B in salt-treated plants. However, it is clear from the results of the present study that foliar-applied Se, B and Se+B mitigated the salt stress by modulating the antioxidant defense system, ROS metabolism and glyoxalase system (Figure 6).

## 4. Materials and Methods

### 4.1. Plant Material and Treatments

Healthy, matured, well-dried and uniform soybean (*Glycine max* cv. BINA Soybean-5) seeds were sown in plastic pots (14 L). Organic manure, urea, triple super phosphate and muriate of potash were applied as basal dose without B. At 20 days after sowing (DAS) along with control (0 mM NaCl), mild, moderate and severe salinity was imposed on plants by treating with 150, 300 and 450 mM NaCl, respectively. Single and combined supplementation of Se (50 µM Na_2_SeO_4_) and B (1 mM H_3_BO_3_) was accomplished at 16, 20, 24 and 28 DAS under control and saline conditions. The experiment was laid out in completely randomized design (CRD) with three replications.

### 4.2. Growth Parameters

For measuring plant height five plants were selected from each replication, and height was taken from the ground level to the tip of the plant. The average height of five plants was considered as the height of the plants for each replication and expressed as cm.

From each replication randomly three sample plants were uprooted, then roots were separated and shoots were weighed in a balance and after that averaged the weight to measure shoot FW plant^−1^_._

After measuring shoot FW, shoot samples were oven-dried at 80 °C for 48 h, then weighed. The average DW was measured and considered as the shoot DW plant^−1^.

For leaf area measurement, leaf images were taken by a digital camera and the area was calculated using Image-J software v. 1.8.0 [50].

### 4.3. Leaf Relative Water Content and Proline Content

Leaf relative water content was measured according to Barrs and Weatherly [51]. After collecting fresh leaves, FW was measured and then dipped into distilled water for 8 h. After that, turgid weight (TW) was measured and followed by oven drying for 48 h at 80 °C to measure DW. The RWC was calculated from the following equation,

RWC (%) = (FW − DW)/(TW − DW) × 100

Proline content was measured from leaf sample according to Bates et al. [52]. Leaf samples were homogenized by using sulfosalicylic acid, followed by centrifuging at 11,500× *g.* Then glacial acetic acid and acid ninhydrin was added with the supernatant. After cooling the mixture, toluene was added to separate ninhydrin pro-complex and then optical density of the chromophore was observed spectrophotometrically at 520 nm. Finally, Pro content was measured by comparing with a standard curve of known concentration of Pro. The Pro content was expressed as μmol g^−1^ FW.

### 4.4. Determination of H_2_O_2_ Content and Lipid Peroxidation

According to Yang et al. [53], leaf samples of 0.5 g were homogenized by trichloroacetic acid (TCA) and centrifuged at 11,500× *g*. Then, supernatant was mixed with potassium-phosphate (K-P) buffer (pH 7.0) and potassium iodide (KI). After that, H_2_O_2_ content was determined spectrophotometrically by measuring the optical absorption of supernatant at 390 nm by using an extinction coefficient of 0.28 μM^−1^ cm^−1^ which was expressed as nmol g^−1^ FW.

By following the method of Heath and Packer [54] lipid peroxidation was assayed as MDA content which was measured spectrophotometrically at 532 and 600 nm on the basis of thiobarbituric acid reactive substances (TBARS) production by using an extinction coefficient of 155 mM^−1^ cm^−1^ and expressed as nmol g^−1^ FW.

### 4.5. Protein Determination

Protein content was estimated by following the method of Bradford [55] in which a standard curve was prepared from a known concentration of bovine serum albumin (BSA) and used to determine the protein content.

### 4.6. Determination of Enzyme Activities

The APX (EC: 1.11.1.11) activity was estimated spectrophotometrically at 290 nm according to the method of Nakano and Asada, [56]. The solution of K-P buffer (pH 7.0), reduced ascorbate (AsA), H_2_O_2_, ethylenediaminetetraacetic acid (EDTA) and extract for enzyme were used during the process where 2.8 mM^–1^ cm^–1^ was the extinction coefficient and it was expressed as μmol min^−1^ mg^−1^ protein.

The MDHAR (EC: 1.6.5.4) activity was determined from the reaction mixture consisting of Tris-HCl buffer (pH 7.5), AsA, ascorbate oxidase (AO), nicotinamide adenine dinucleotide phosphate (NADPH) along with enzyme and 6.2 mM^–1^ cm^–1^ was the extinction coefficient [57]. The activity of MDHAR is expressed as nmol min^−1^ mg^−1^ protein.

The DHAR (EC: 1.8.5.1) activity was measured spectrophotometrically by following the method of Nahar et al. [57] at 265 nm. The reaction mixture consisted of K–P buffer (pH 7.0), reduced glutathione (GSH), dehydroascorbate (DHA), EDTA and enzyme solution. Extinction coefficient 14 mM^–1^ cm^–1^ was used during the enzyme calculation and DHAR activity was expressed as nmol min^−1^ mg^−1^ protein.

The GR (EC: 1.6.4.2) activity was determined from the reaction of the reaction mixture which consists of K-P buffer (pH 7.0), oxidized glutathione (GSSG), EDTA, NADPH and enzyme solution at 340 nm using 6.2 mM^–1^ cm^–1^ as the extinction coefficient [22]. The GR activity was expressed as nmol min^−1^ mg^−1^ protein.

The CAT (EC: 1.11.1.6) activity was estimated according to Hasanuzzaman et al. [22] at 240 nm and read from a reaction mixture of K-P buffer (pH 7.0), H_2_O_2_ and enzyme solution and computed using 39.4 M^–1^ cm^–1^ as the extinction coefficient and expressed as μmol min^−1^ mg^−1^ protein.

The activity of GST (EC: 2.5.1.18) was measured from the reaction mixture of GSH, 1-chloro-2, 4-dinitrobenzene (CDNB), Tris-HCl buffer (pH 6.5) and enzyme extract, using 9.6 mM^–1^ cm^–1^ as the extinction coefficient [22]. The GST activity was expressed as nmol min^−1^ mg^−1^ protein.

The GPX (EC: 1.11.1.9) activity was read from the absorbance of the reaction between K-P buffer (pH 7.0), GSH, EDTA, sodium azide (NaN_3_), NADPH, GR, H_2_O_2_ and enzyme at 340 nm. Extinction coefficient 6.62 mM^–1^ cm^–1^ was used during the calculation of enzyme activity [57]. The GPX activity was expressed as nmol min^−1^ mg^−1^ protein.

The POD activity (EC: 1.11.1.7) was determined according to Hemeda and Klein [58], at 470 nm guaiacol oxidation was observed spectrophotometrically. The reaction buffer contained sodium phosphate buffer (pH 6.0), methoxyphenol and 30% H_2_O_2_. The activity was computed by using an extinction coefficient of 6.58 mM^−1^ cm^−1^ and it was expressed as μmol min^−1^ mg^−1^ protein.

The Gly I (EC: 4.4.1.5) activity was read from the reaction of the mixture which consists of K-P buffer (pH 7.0), GSH, magnesium sulfate and MG at 240 nm by using 3.37 mM^–1^ cm^–1^ as the extinction coefficient [22]. The Gly I activity was expressed as μmol min^−1^ mg^−1^ protein.

The Gly II (EC: 3.1.2.6) activity was measured from the reaction of enzyme, Tris-HCl buffer (pH 7.2), 5, 5′-dithiobis-2-nitrobenzoic acid (DTNB) and *S*-D-lactoylglutathione at 412 nm by using an extinction coefficient of 13.6 mM^–1^ cm^–1^ [22]. The Gly II activity was expressed as μmol min^−1^ mg^−1^ protein.

### 4.7. Statistical Analysis

All data of three replications were statistically analyzed by using CoStat v.6. 400 [59]. Data were analyzed in one-way analysis of variance (ANOVA). The mean difference was compared by Fisher’s least significant difference (LSD) test with the 5% level of significance.

## 5. Conclusions

The results of the present study clearly reveal that single and combined supplementation of Se and B mitigated the deleterious effect of salt stress and salt-induced oxidative stress in soybean, and also improved salt tolerance. However, exogenous Se, B and Se+B declined the salt-induced oxidative stress in soybean by upregulating the enzymatic activities of the antioxidant defense system and glyoxalase system, ultimately by diminishing the ROS accumulation which was indicated by the reduced H_2_O_2_ content and MDA content. Moreover, growth parameters and leaf RWC were also improved due to supplementation of Se+B under salt stress. Though in the present study, Se and B alone showed similar results to the combined application, but it was evident that the combined application of Se+B was more effective than a single application to confer the different levels of salt stress. Therefore, further investigations should be performed to ascertain the doses of Se, B and Se+B under different levels of salinity to mitigate the salt stress, as a high concentration of Se and B is phytotoxic. The role of B in MG detoxification has rarely been studied, and the mechanism is not well known yet; thus, it needs advanced future investigations.

## Data Availability

All data are presented in this manuscript.

## Abbreviation

AO−ascorbate oxidase; APX−ascorbate peroxidase; AsA−ascorbate; BSA−bovine serum albumin; CAT−catalase; CDNB−1-chloro-2, 4-dinitrobenzene; DHA−dehydroascorbate; DHAR−dehydroascorbate reductase; DTNB−5, 5′-dithiobis (2-nitrobenzoic acid); EDTA−ethylenediamine tetraacetic acid; Gly I−glyoxylase I; Gly II−glyoxalase II; GPX−glutathione peroxidase; GR−glutathione reductase; GSH−glutathione; GSSG−oxidized glutathione; GST−glutathione *S*-transferase; KI−potassium iodide; MDA−malondialdehyde; MDHAR−monodehydroascorbate reductase; MG−methylglyoxal; NADPH−nicotinamide adenine dinucleotide phosphate; POD−peroxidase; Pro−proline; ROS−reactive oxygen species; TBARS−thiobarbituric acid reactive substances; TCA−trichloroacetic acid.
